# Complementary Transcriptome and Proteome Analyses Provide Insight into the Floral Transition in Bamboo (*Dendrocalamus latiflorus* Munro)

**DOI:** 10.3390/ijms21228430

**Published:** 2020-11-10

**Authors:** Xiaoyan Wang, Yujiao Wang, Guoqian Yang, Lei Zhao, Xuemei Zhang, Dezhu Li, Zhenhua Guo

**Affiliations:** 1 Germplasm Bank of Wild Species, Kunming Institute of Botany, Chinese Academy of Sciences, Kunming 650201, China; wangxiaoyan@mail.kib.ac.cn (X.W.); wangyujiao@mail.kib.ac.cn (Y.W.); guoqian.yang@sjtu.edu.cn (G.Y.); zhaolei@mail.kib.ac.cn (L.Z.); zhangxuemei@ynutcm.edu.cn (X.Z.); 2 Kunming College of Life Science, University of Chinese Academy of Sciences, Kunming 650201, China; 3 School of Agriculture and Biology, Shanghai Jiao Tong University, Shanghai 200240, China; 4 Yunnan Key Laboratory for Dai and Yi Medicines, Yunnan University of Chinese Medicine, Kunming 650500, China

**Keywords:** floral transition, iTRAQ, biomarker gene, mRNA-protein correlation, bamboo

## Abstract

Most woody bamboos bloom only once after long vegetative growth phases and die immediately afterwards. It is difficult, however, to determine the timing of the floral transition, as little information is available on the molecular mechanism of plant maturity in bamboos. To uncover the bamboo floral transition mechanism, its morpho-physiological characteristics, transcriptomes and large-scale quantitative proteomes were investigated in leaves which were collected at different stages during floral transition in a woody bamboo, *Dendrocalamus latiflorus.* We identified many flowering time-associated genes and the continued increase and decrease genes were screened as flowering biomarker genes (e.g., the *MADS14* and *bHLH13* genes). These different genes were assigned to specific metabolic pathways by the Kyoto Encyclopedia of Genes and Genomes (KEGG). And the photoperiod pathways depending on the circadian rhythm may play an essential role in the bamboo floral transition. In addition, a total of 721 differently expressed proteins of leaves from the vegetative-to-reproductive stages were identified. Fifty-five genes were specifically differentially expressed at both the transcriptomic and proteomic levels, including genes related to photosynthesis and nucleotide sugar, which may be involved in the floral transition. This work provides insights into bamboo flowers and the management of forest breeding.

## 1. Introduction

All organisms go through a series of distinct developmental phases during their growth [1]. The transition to flowering is a critical step in these developmental phases. The genetic and biochemical routes that provide the cues that influence flowering are often referred to as flowering pathways and include vernalization and autonomous pathways; photoperiod pathways and the circadian clock; the gibberellin pathway; the ambient temperature pathway; and the age pathway [2,3,4]. Multiple floral stimuli eventually move from the leaves to the shoot apical meristem (SAM), a group of stem cells in the shoot apex, where they are converted into local cues that evoke the developmental phase transition [5].

Bamboos can be classified into three categories based on their flowering behavior: species that flower annually, species that flower gregariously and periodically, and species that flower irregularly [6]. Most woody bamboos belong to the second category [7,8] and use a semelparity strategy, wherein they flower once and die at the end of 3–120 years (or longer) of vegetative growth phases [9]. Many bamboos have a special life history trait of monocarpic mass flowering and death, suggesting a special genetic mechanism controlling floral transition in bamboos. This collective death of bamboos results in considerable loss of forest agencies and private cultivators and has produced serious ecological crises, most strikingly for giant pandas [10]. Systematic theories on the bamboo flowering mechanism have not yet been in development, and the studies already done mostly focus on discussing the behaviors of flowering and analyzing of genes’ roles in flowering [11,12,13]. With the development of high-throughput sequencing (including RNAseq) technology, many genes involved in bamboo flowering have been identified [11,12,13]. However, to date, studies have been done primarily on flower development, but little has been investigated on the floral transition in bamboos.

In model plants and other non-model plant species with large and non-sequenced genomes, transcriptome and proteome profiling are powerful methods to analyze dynamic changes in multiple biological processes. Transcriptomic and proteomic analyses are extremely efficient methods for identifying differential expression genes at the whole-genome level. The developed isobaric tags for relative and absolute quantitation (iTRAQ) technology was proven to be very efficient in protein profiling [14]. However, because of the length of a bamboo’s special flowering mechanism phase, it is difficult to obtain this material. Our present study found the *Dendrocalamus latiflorus* as an ideal bamboo species. *D. latiflorus* (Bambuseae, Bambusoideae, Poaceae), as a woody bamboo, is the most widely distributed and cultivated grass in southern China [15]. The biological characteristics of flowering and the regeneration process of *D. latiflorus* were investigated from 2008 to 2012. In this study, a clump of *D. latiflorus* flowered when the mother culms died, followed by the surviving rhizome system; a few culms continued to flower each July to October over the next three years. To determine the mechanism of the floral transition of bamboos in the same flowering culms, we cataloged the differences in the abundance of mRNAs and protein by the integrated profiling of gene activity using RNA-seq and iTRAQ. Comparisons of transcriptomic and proteomic data provided novel indications as to which processes matching the floral transition are regulated at the level of the transcriptome and which are controlled at the proteome level. Transcriptome and proteome profiling will provide a supplement for the genome resources of *D. latiflorus*. This study aims to identify a set of candidate genes and pathways associated with the floral transition in bamboo. Our findings are expected to outline the mechanism of flowering, thereby overcoming a barrier for conventional bamboo propagation technology.

## 2. Results

### 2.1. Leaf Microstructure and Chlorophyll Fluorescence of D. latiflorus

**Figure 1:**
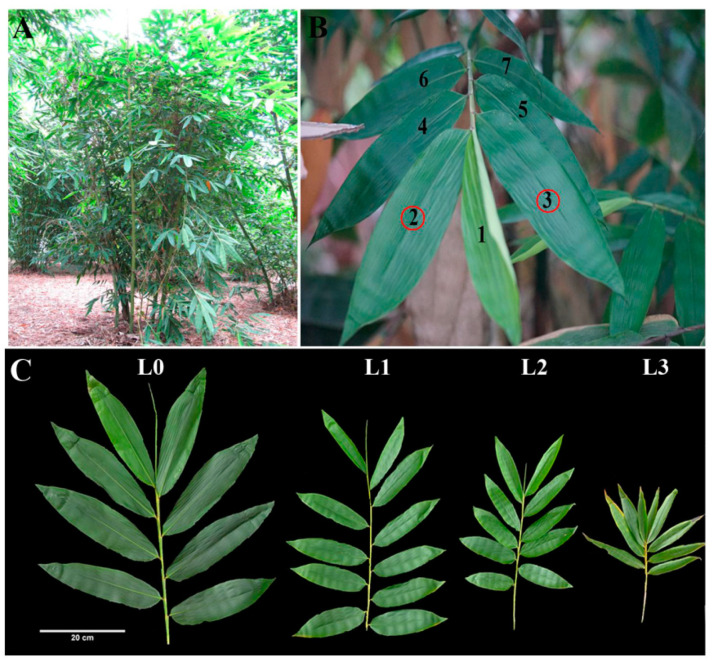
Tissues sampled for RNA-seq and protein analyses of *D. latiflorus*. (**A**) External morphology of *D. latiflorus*, a cluster-growing bamboo, shown in situ. (**B**) Leaves were selected from the 2nd and 3rd knob of the branch apex. (**C**) The branches of *D. latiflorus* can be divided into four growth stages during floral transition, including the large foliage leaves (L0), small foliage leaves (L1), large flowering leaves (L2), and small flowering leaves (L3). L0 belongs to the vegetative clumps. L1, L2, and L3 belong to the same reproductive ramets with at least one pole blooming.

We collected leaves from four stages, including leaves of vegetative clumps (L0) and leaves from flowering clumps (L1, L2, L3) (Figure 1). Leaves from flowering clumps can be divided into three periods: leaves from non-flowering clumps (L1) and big and small leaves from flowering clumps (L2, L3).

**Figure 2:**
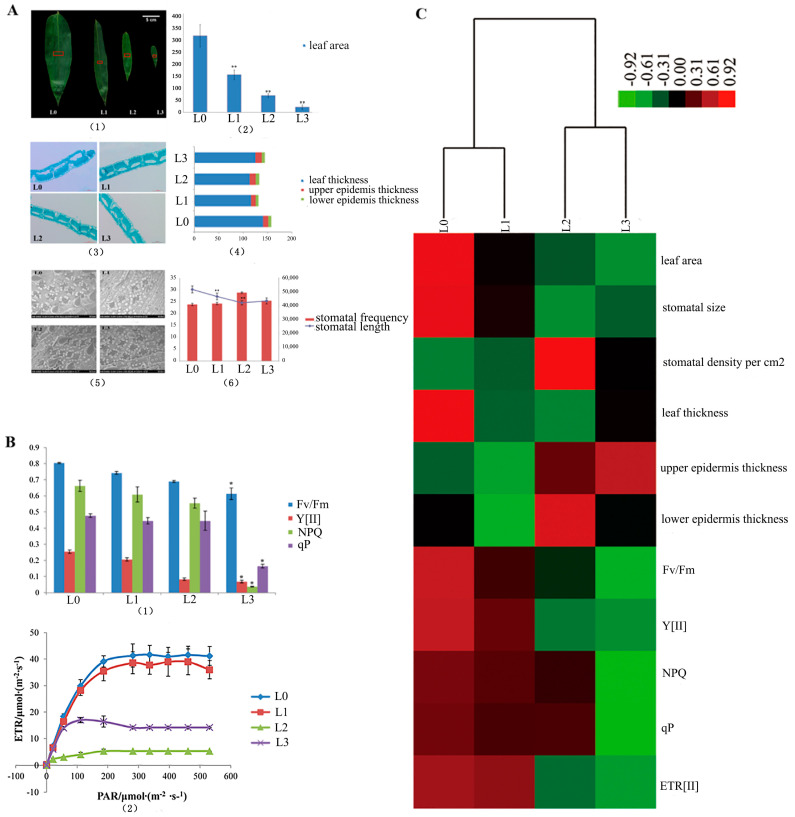
The statistics of the leaf surface, stomata and chlorophyll fluorescence. (**A**) (1) The middles of the leaf blades were examined by their anatomy (indicated by the red square); (2) leaf area from L0 to L3; (3) SEM of L0, L1, L2 and L3; (4) stomatal frequency and stomatal length from L0 to L3; (5) the leaf anatomy (6) the blade thickness, epicuticle thickness, and lower epidermis thickness. (**B**) The parameters of chlorophyll fluorescence of the flowering leaves of *D. latiflorus*. (1) the parameters of chlorophyll fluorescence from L0 to L3; (2) The curves of electron transfer reactions (ETR) from L0 to L3. (**C**) The heat map of the measurement parameters from L0 to L3. ** *p* < 0.01, significant difference; * *p* < 0.05, difference by *t*-test.

The surface area of the blade and the stomata in the middle cross-sectional blade were calculated and studied (Figure 2(A1)). The leaf area significantly decreased from L0 to L3 (*p* < 0.01, L0 > L1 > L2 > L3, Figure 2(A2)). The transection of the leaf blade is shown in Figure 2(A3). They present the same structure without Kranz anatomy in the four stages. In Figure 2(A4), the blade thickness of L0 is significantly thicker than that of L1, L2 and L3 (*p* < 0.01). The epicuticle thickness of L1 is significantly thicker than that of L0, L2 and L3 (*p* < 0.01). The thickness of the blade, epidermis and epicuticle showed no differences in the L2 and L3 (*p*>0.05). The comparative morphology of the leaf stomata by SEM is displayed in Figure 2(A5). This illustration shows that the mean stomatal length in the vegetative clumps (L0) was significantly longer (*p* < 0.01) than that of L1, L2 and L3 in the flowering clumps. However, there were no significant differences (*p* > 0.05) between L2 and L3 (Figure 2(A6)). The stomata density per square centimeter of the leaf surface in the vegetative clumps (L0) is almost the same as that in the flowering clumps (L1, L2 and L3). In the present study, the parameters of chlorophyll fluorescence (Fv/Fm, Y[II], NPQ and qP) showed significant differences between L0 and L3 (*p* < 0.01) (Figure 2(B1)). The Fv/Fm values of L1, L2 and L3 were lower than 0.8, which indicated plant stress. The curves of electron transfer reactions (ETR) from L0 to L3 were changed with light intensity (Figure 2(B2)). It showed the linear relationship between ETR and photosynthetic active radiation (PAR). ETR rapidly increased with the increase in light intensity and was gradually saturated at 300 μmol/(m^2^·s). The electron transfer ability of L0 and was strongest in the same local environment. The L0 value of ETR was the highest, followed by L1, L2 and L3.

The measurements of the parameters from L0 to L3 were clustered by heat map (Figure 2C). The hierarchically clustered heat map showed the relationship in the development of the morpho-physiological characteristics of the leaves at different stages during floral transition. Here, L0 and L1 show similar developmental characteristics and L2 and L3 show similar developmental characteristics.

### 2.2. De Novo Assembly and Functional Annotation of the Differentially Expressed Genes (DEGs)

In this study, 12 cDNA libraries (four stages, with three biological repeats) were constructed from total RNA of L0, L1, L2 and L3, and were pair-end sequenced using the Illumina Hiseq^TM^ 2000 platform (Illumina, San Diego, CA, USA). In total, 207.68 million paired-end reads were generated. After cleaning and quality checks, the reads were assembled into contigs using Trinity. A final set of 155,494 unigenes was obtained, with an average size of 1477 bp, and N50 of 2069 bp (Table 1). The assembly produced a substantially large number of unigenes and 125,293 unigenes were more than 1000 bp in length (Supplementary Materials Figure S1). We mapped our RNA-seq reads back to the constructed reference sequences, 80% of which were mapped to the reference sequences (Table S1).

**Figure 3:**
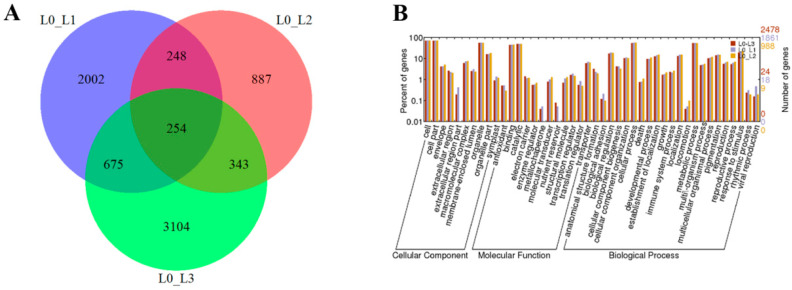
Numbers of shared and unique genes in L0 vs. L1, L0 vs. L2 and L0 vs. L3 (**A**) and the gene ontology classification of L0 vs. L1, L0 vs. L2 and L0 vs. L3 (**B**).

The DEGs in L0 vs. L1, L0 vs. L2 and L0 vs. L3 were identified as 3179, 1732 and 4376, respectively (Figure 3A). To predict and analyze the function of the DEGs, we used four databases, including NR, UniprotKB, GO and KEGG (Table S2). For the L0 vs. L1, L0 vs. L2 and L0 vs. L3, we found that a total of 3045, 1670 and 4128 (95.8%, 96.4% and 94.4% of all unigenes, respectively) unigenes provided significant BLAST results in NR based on their sequence homologies. According to GO classification, a total of 1861 and 2478 sequences (58.5% and 56.6% of all the unigenes) were categorized into 44 functional groups in the L0 vs. L1 and L0 vs. L3. However, a total of 988 unigenes (57% of all the unigenes) were categorized into 41 functional groups in the L0 vs. L2. The functions of the extracellular region, metallochaperone and nutrient reservoir were not found in the L0 vs. L2 (Figure 3B).

To further test the reliability of the results from the next generation sequencing platform, a quantitative real-time PCR (qRT-PCR) analysis was performed on 18 of the differentially expressed transcripts (Table S3). The expression patterns of 17 genes detected by qRT-PCR fit well with those from the RNA-seq results, except the unigene 33763 (Figure S2).

### 2.3. Screen Candidate Marker Genes

**Figure 4:**
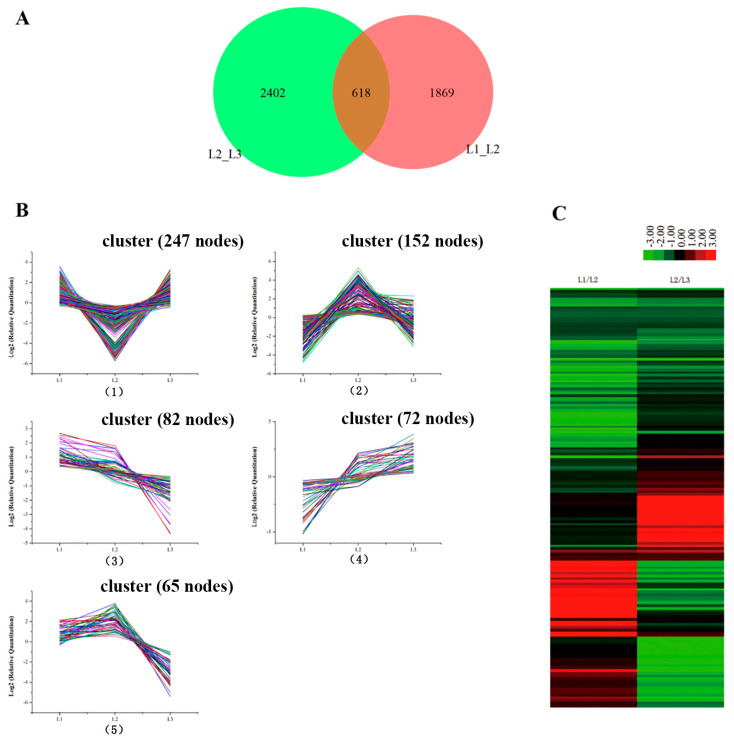
The differently expressed genes from L1 to L3 in the same bamboo clump. (**A**) A Venn diagram showing the number of differentially expressed genes between every two samples and the number of joint differentially expressed genes. (**B**) The clustering of differentially expressed genes. Expression ratios are expressed as Log2. The 8 major clusters obtained by the K-means algorithm, representing (1) those down-regulated at L2 and up-regulated at L3; (2) those up-regulated at L2 and down-regulated at L3; (3) those down-regulated from L1 to L3; (4) up-regulated from L1 to L3; and (5) those up-regulated at L2 and down-regulated at L3; the expression level at L3 is lower than that at L1. (**C**) Hierarchical clustering of the differentially expressed genes.

Leaves from L1 to L3 were in the culms of the same bamboo flowering clump (sympodial). The gene expression profiles showed 618 genes that were the common DEGs during floral transition, as illustrated in Figure 4A. The 618 candidate genes were determined by a cluster analysis based on the k-means method [16]. Five expression patterns (clusters) of the 618 DEGs were identified (Figure 4B). Cluster 1 was the most abundant group and contained 247 genes that down-regulated at L2 and then up-regulated at L3. Clusters 2 and 5 consisted of 152 and 65 genes, respectively, whose expression was up-regulated at L2 and then down-regulated at L3. The differences in the gene expression levels of cluster 5 at L3 were lower than of cluster 2 at L1. Cluster 3 was composed of 82 genes that showed a continuous down-regulation during floral transition. Cluster 4 contained 72 genes which revealed a continuous up-regulation from vegetative growth to reproductive growth.

Hierarchical cluster sample analysis provided an overview of 618 unigenes (Figure 4C). We focused on the continued decreases (82 genes) and increases (72 genes) of the genes during floral transition (from L1 to L3). Of these genes, we chose the 18 genes that had significant biological functions as potential biomarkers (Table S4). The functions of some genes (unigene 138210, unigene 43312 and unigene 29008) were focused on loosening an extension of the plant cell walls by disrupting non-covalent bonding between cellulose microfibrils. Genes specifically involved in regulating floral architecture and plant development (unigene 10723 and unigene 114865) displayed up-regulation. The unigene 39652 coded MADS 15 protein transcript level was extremely low in the foliage leaves L1. Compared to foliage leaves (L1), the expression of the chloroplast protein (unigene 41907: Far1-related sequence 5; unigene 13665: Serine/threonine-protein kinase STN8, chloroplastic; unigene 14850: Transcription factor bHLH13) was down-regulated in flowering leaves (L2 and L3). It is consistent with the results of the photosynthetic rate decrease by the chlorophyll fluorescence. In addition, the expression level of unigene 99180, which is involved in the regulation of the stomatal aperture, showed a continued decrease from L1 to L3, which is consistent with the results in Figure 2(A5). The expression levels of stress-related genes, such as unigene 38555, promoted plant stress tolerance such as heat, chilling, salinity and toxicity; unigene 72242-inactive ADP-ribosyltransferase that functions with SRO1 to regulate oxidative stress, hormonal and developmental responses; unigene 27191, which may be involved in oxidative stress response, displayed a decline during the floral transition. In addition, the levels in the genes modulating plant transpiration efficiency by controlling stomatal density (unigene 133805, unigene 99180) decreased during bamboo flowering. The gene levels specifically involved in sugar transport protein (uniegene 21870) displayed obvious down-regulation.

### 2.4. Identifying D. latiflorus Flowering Time-Associated Genes and Senescence-Associated Genes

We screened the flowering time-associated genes which were assigned into different pathways [4] (Figure S3). These genes consisted of an autonomous pathway, a vernalization pathway, a photoperiod pathway, a gibberellin pathway and an aging pathway. The main genes associated with flowering time are displayed in Table 2. These genes included floral integrator pathway genes such as *FT*, *SOC1* and *AGL24*; the vernalization pathway genes related to Frigida (*Fri*) and Vernalization-insensitive 3 (*VIN3*); the autonomous pathway genes *FCA* and *FY*; the gibberellin pathway gene *GID1*; the floral meristem identity gene *MADS14*; the aging pathway gene *SPL9*; and other flowering-related genes *AGL6* and *EMF1*. All these were identified in our *D. latiflorus* RNA-seq database. Most of these genes showed down-regulation at L3 compared to L0 (Table 2). Some senescence-associated genes were dominantly expressed in the reproductive tissue L3. Of them, the FPKM value of Unigene 25575 demonstrated higher expression levels than those of other genes, with the highest observed in L3 among the four stages (Figure S4).

### 2.5. Pathways Involved during Floral Transition

We focused on the DEGs in the L0 vs. L3. In total, 1719 sequences (39.3% of all the unigenes) were assigned to 285 KEGG pathways. The pathways with the greatest representation by unigenes were metabolic pathways, genetic information processing and signaling, and cellular processes. We found 55 unigenes involved in plant hormone signal transduction, which contained 8 pathways (Figure S5, Table S5). The photoperiod pathways, depending on the circadian rhythm, were inferred. Several circadian clock-controlled genes (16 unigenes, 0.93%) regulating photoperiodic flowering in *D. latiflorus* were found in the pathway.

**Figure 5:**
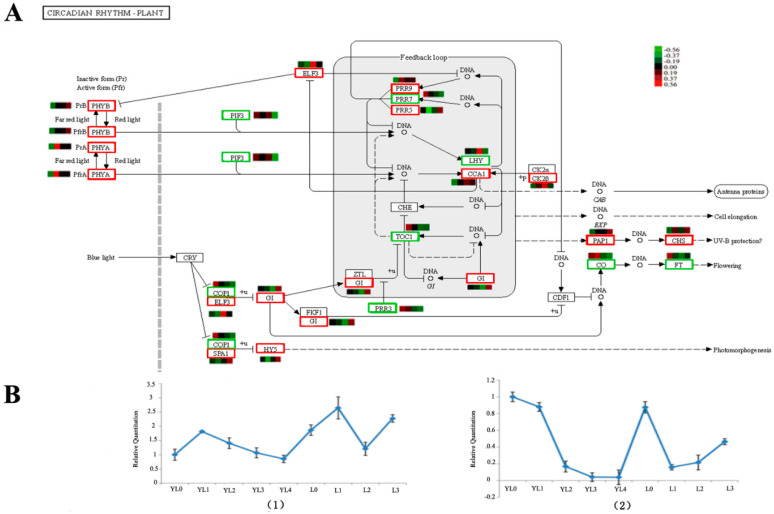
The photoperiod pathways depended on the circadian rhythm for the unigenes according to KEGG annotation (**A**) and the actual expression levels *CO*/*FT* (**B**) in different stages of the bamboo. (1) The *CO* gene expression level; (2) the *FT* gene expression level. The red square in (**A**) indicates up-regulation in L3 relative to that in L0, the green square indicates down-regulation in L3 relative to that in L3, and the bar in (**B**) denotes the standard error. YL0 shows 3 months of bamboo seedlings, YL1 shows one year of bamboo seedlings, YL2 shows two years of bamboo seedings, YL3 shows five years of bamboo seedlings, YL4 shows six years of bamboo seedlings. L0, L1, L2 and L3 have the same meaning in this paper.

In the metabolic pathway dependent on the circadian rhythm regulating flowering (Figure 5A), some key genes (Table 3) were observed to be up-regulated in L3, such as the Two-component response regulator-like APRR5 (*PRR5*), which exhibited 10-times higher expression than that in L0. We collected bamboo seedlings of different ages (Figure S6). The actual expression level of *CO* and *FT* were performed (Figure 5B); the *CO* gene expression was more up-regulated at YL1 compared to YL0, and then down-regulated from YL1 to YL4. It showed a higher expression level at L3 than that at L0 in the adult phase. *FT* gene expression was down-regulated from YL0 to YL4 and up-regulated at L0; then, it continuously rose from L1 to L3, and the expression level at L3 was lower than that at L0. In addition, the unigenes mentioned in the article were listed in Table S6.

### 2.6. Proteomics Analysis

**Figure 6:**
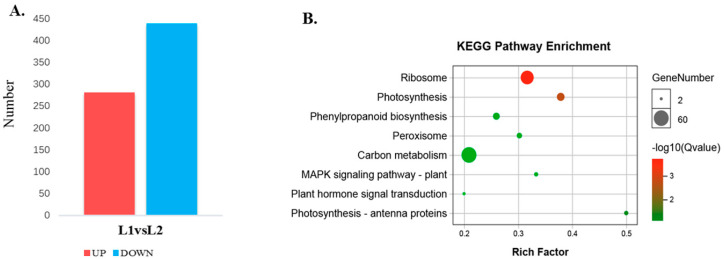
Different expression proteins in L1 and L2 and the KEGG pathway enrichment. (**A**) The statistics of different expression proteins in L1 and L2; (**B**) the KEGG pathway enrichment analysis of the different expression proteins.

A total of 4636 proteins were identified and protein annotation was performed by BLAST against Nr, Uniprot, KEGG and COG. Of these 4629 annotated proteins, 4282 protein sequences were classified into 24 COG categories (Figure S7, Table S7). A 95% confidence level (*p <* 0.05) and a 1.2-fold change were used to identify proteins that were differentially expressed between L1 and L2 during floral transition. Using these criteria, 721 differentially expressed proteins (282 up-regulated and 439 down-regulated) were found (Figure 6A, Tables S8 and S9). The 721 differentially expressed proteins were searched against the KEGG pathway with 397 proteins significantly enriched (*p <* 0.05) in 8 metabolic pathways (Figure 6B). Notably, 44 differential proteins were predicted to be involved in photosynthesis-related pathways (photosynthesis: 29; 5.1%; photosynthesis-antenna proteins: 15; 2.64%) during floral transition. Many proteins were mainly focused on the “ribosome”, “phenylalanine metabolism”, “peroxisome”, etc.

### 2.7. Integrative Analysis of the Proteome and Transcriptome of L1 and L2

The distribution of the corresponding mRNA: protein ratio is shown by a scatterplot analysis of the log_2_-transformed ratios (Figure S8). The Pearson correlation coefficients for these data were 0.48 (*p* < 0.05). This result indicates that the correlation between the transcriptome and proteome was weak during floral transition.

There were fifty-five identified proteins that had high corresponding transcripts in the RNA-seq data (Table S8), and these genes were differentially expressed at the transcriptomic and proteomic levels. There were, in total, 3 genes in the photosynthesis pathway (Table 4), including 2 down-regulated genes, such as *PsbS* (Unigene124766) and *PsaH* (Unigene62245) and 1 up-regulated gene, such as *psbP* (Unigene36630). In the carbon metabolism pathway, the NADP-dependent malic enzyme (NADP-ME, Unigene9416) was extremely up-regulated. Chlorophyll content and photosystem II activity were inversely correlated with the level of NADP-ME activity. These results suggest that the different photosystem between L1 and L2 may be caused by excessive NADP-ME activity. Unigene117107, Unigene148716 and Unigene111502 were down-regulated and belonged to the amino sugar and nucleotide sugar metabolism pathway. Unigene129955, a vegetative storage protein was also down-regulated.

## 3. Discussion

Bamboo flowering is a highly coordinated, genetically programmed process with a change in leaf size noted a peculiar phenomenon. Little is known about the mechanisms responsible for floral transition and genomic information for *D. latiflorus*. According to the morphological leaf formed during floral transition in *D. latiflorus*, the developmental processes were divided into four stages (L0, L1, L2 and L3), and RNA-seq uncovered the four development stages of the leaf transcriptome landscape. The available transcriptomic data for *D. latiflorus* met the initial information needs for functional studies of this species and its relatives. Technological advances, particularly mass spectrometry and high-throughput cell imaging, have allowed for large-scale surveys of the proteome [17]. This study of the leaf anatomical structure and chlorophyll fluorescence in *D. latiflorus* (Figure 2C) demonstrated that these characteristics in L1 are different from those in L2. The floral transition from L1 to L2 is a critical point. We selected the L1 and L2 in the same flowering clumps with the same biological background to study proteomic characterization by iTRAQ during the floral transition; a supplement for the description of the transcriptome. Both the transcriptomic and proteomic data are important for deciphering the molecular processes involved in floral transition.

### 3.1. Identifying the Genes Associated with D. latiflorus Flowering Time

From all the identified genes, the genes regulating flowering time in the most important pathway are shown in Table 2. Of these pathways, genes *FRI* (unigene 57034) and *VIN3* (unigene 110221) are known to be vernalization. FRI is a floral repressor and plays a key role in the Arabidopsis flowering time. The dominant alleles of *FRI* confer late flowering, which is reversed to earliness by vernalization. The loss-of-function mutation at *FRI* provided the basis for the evolution of many early-flowering ecotypes [18]. In this study, we found that *FRI* gene expression levels were higher in L0 (Vegetative state) than in L3 (Reproductive state). This finding agrees with previous results from *Phyllostachys violascens* showing that *PvFRI* was expressed in all tested organs with higher expression in non-flowering than flowering plants [19].

In this study, the genes associated with the gibberellin pathways included the genes *GID1* (*Gibberellin receptor GID1*, Unigene132606) and *GA20ox* (*Gibberellin 20 oxidase 1-B*, Unigene33012). Gibberellins (GAs) consist of a large group of tetracyclic diterpenes, a few of which are endogenous growth regulators and play roles in plant growth and development [20]. GID1 is a key mediator of GA response pathways. By binding to a nuclear receptor, *GID1* gibberellins regulate gene expression by promoting degradation of the transcriptional regulator DELLA proteins, including gibberellin insensitive (GAI) protein [21]. Mutant and expression analyses demonstrated that enzymes catalyzing the early steps in the GA biosynthetic pathway are mainly encoded by single genes, while those for later steps (i.e., *GA20ox*) are encoded by gene families.

We also identified the genes *FCA* and *FY*, which belong to the autonomous pathway. *FCA* and *FY* are involved in one sub-pathway, and interact to regulate the RNA processing of *FLC* in the *Arabidopsis* autonomous pathway. In addition, the sequence homologs for AGAMOUS-LIKE 6 (*AGL6*) and *EMF1* were found in our database. AGL6 genes are MADS-box genes expressed in floral tissues, which regulate floral organ identity and floral meristem determinacy [22]. *EMF1* is involved in delaying both the vegetative to reproductive transition and flower initiation in Arabidopsis [23]. The vegetative phase change is initiated by a decrease in the expression of miR156 and consequent increases in the expression of *SBP* genes, i.e., the paralogous *SPL9* gene in newly formed organs [24]. The *SPL* gene expression in L0 was higher than that in L3 in the present study. Consistent with this finding, we discovered that the size in L0 is significantly larger than in L3 by the quantitative statistics of bamboo leaf size (Figure 2A).

### 3.2. Identification of the Flowering Integration Genes and Metabolism Pathways Involved in Floral Transition

In this study, three integration genes, *SOC1*, *AGL24* and *FT*, all of which exist at the point of convergence in several flowering-time pathways, were identified. The gene structure of *SOC1* is significantly different between woody and herbaceous bamboos, which is potentially the cause of the long vegetative stage in the lifecycle of woody bamboos [25]. *AGL24* plays a role in the regulation of flowering time, which is a promoter of flowering and acts as a positive regulator of *SOC1* [26]. In bamboo, recent research has found *PvFT1*, which is a candidate gene for florigen, and the expression of *PvFT1* reached its highest level 20 to 30 days before flowering in the leaves [11]. In the present study, compared with L0, the expression level of *FT* genes showed a decline in L3 (Table 2). We conclude that the FT protein moves into the SAM during the floral transition in *D*. *latiflorus*. Future studies of floral transition in bamboo may find this to be a crucial candidate gene.

We focused on the photoperiod pathway that depends on the circadian clock (Figure 5). The photoperiod pathway controls this response in the leaves through a signaling cascade involving *Digantea* (*GI*) and the transcriptional regulator *CO* and *FT* gene [27]. Timing of cab expression 1 (*TOC1*), late elongated hypocotyl (*LHY*) and circadian clock associated 1 (*CCA1*) are the parts of the central mechanism generating circadian rhythms in plants [28]. *LHY* and *CCA1* were proposed to act along with *TOC1* in a transcriptional–translational negative feedback loop [29]. The photoperiodic genes also play an important role in bamboo flowering [30]. This study elucidated the photoperiodic regulation of bamboo homologs of important flowering genes. The sequences homologues for genes involved in regulation of the circadian clock described above were listed in Table 3. The main DEGs in the L3, depending on the circadian rhythms regulating flowering during floral transition, were up-regulated compared to those in the L0.

The long juvenile phase of bamboo is not obviously regulated by day length. To determine whether the bamboo *CO*/*FT* ortholog is also involved in the regulation of flowering time, we analyzed the *CO*/*FT* gene expressions as the core of the pathway that promotes flowering (Figure 5A). We collected leaves from bamboo seedlings aged 3 months to 6 years. The *CO* gene expression levels from YL0 to YL3 and L0 to L1 display a decrease, and may include it acting as repressors of *FT* transcription, or leaf-derived signals such as *FT* are transported to particular meristems [7]. When adult bamboo was in the vegetative state (L0, adult vegetative state), the *FT* gene expression showed a high level compare to juvenile stages. A previous study detected the highest expression level of *FT* homologs in the flowering clumps in bamboo species (*Phyllostachys meyeri* and *Shibataea chinensis*) [31]. This study is consistent with our results, which displayed higher expression in the L3 (reproductive state) than in the L1 (vegetative state) of the same flowering clumps. The *FT* transcript expression displayed a gradual increase as the bamboo grew older from L1 to L3, suggesting that a critical level of *FT* expression is needed to initiate flowering.

In contrast to FT, the CO mRNA cycles, regardless of day length, showed a prominent peak in the night following either a long or short day, and an earlier shoulder in the afternoon only on long days [32]. The CO protein promoted the transition from vegetative growth to flowering [33] and the gene *CO* expression level decreased from YL1 to YL4 during the juvenile stages. This may be why bamboo waits so long to flower. In the same flowering clump, the highest expression level was observed in L1, and then subsequently bloomed. Contrary to the *FT* gene, which the *CO* gene showed to be the lowest expression level in L1, we speculated that the highest expression level was produced by the mobility of the FT protein, however this hypothesis needs further experimentation. Fifteen unigenes containing the conserved CCT domains were identified, and nine genes were used to perform the phylogenetic analysis (Figure S7). The sequence data showed a high similarity with these genes in other homologous species (*Zea mays*, *Setaria italica*, *Hordeum vulgare* and *Brachypodium distachyon*). These are the regions with a higher homology representing units of functional importance.

In the case of floral induction by photoperiod, long-distance signaling is known to occur between the leaves and the shoot apical meristem (SAM) via the phloem [34], including the signaling of hormones (Auxin, Abscisic acid, Cytokinin, Abscisic acid (ABA), Salicylic acid, Ethylene and Brassinosteroid) and sugar (Table S5). This signaling appeared to function as like other key signals in the regulation of bamboo flowering and represents an important resource for the future of floral transition and flower organ development. More studies on these expression patterns and functions will help outline the relevant floral mechanisms.

### 3.3. Screened Floral Biomarker Genes

To better understand the information related to the gene expression of *D. latiflorus*, we analyzed the gene expression patterns under different developmental phases. Our analysis identified 618 genes that are commonly differentially expressed in L1 vs. L2 and L2 vs L3, which potentially affect the floral transition in *D. latiflorus*. We then determined the gene expression profiles in the compared samples. In total, five groups were defined according to their expression profiles. We focused on the gene that showed continuous up-regulation and down-regulation, and could be screened for candidate genes predicting the identity of the bamboo clump that gives rise to a vegetative or a reproductive state. In Figure 2(B2), the Fv/Fm ratio of L3 is significantly lower than that of L1 and showed that the reproductive bamboo was inefficient, with low nutrients and weak photosynthesis [35,36]. These results are consistent with those of RNA-seq, whose expression levels continued to decrease from L1 to L3. The bHLH13 transcription factors function redundantly to negatively regulate Jasmonates-mediated plant defense and development [37]. In the present study, the transcription factor bHLH13 decreased during floral transition. The Jasmonates-related genes may increase from L1 to L3, and then will increase the expression of leaf senescence-associated genes. *MADS14* is induced in the SAM during the meristem phase transition [38]. In our study, the *MADS14* gene expression level increased during floral transition and plays an important role in the phase transition in bamboo. We found that the progression of bamboo flowering is accompanied by the rapid loss of chlorophyll, decreased abundance of photosynthesis-related proteins and an increased expression of senescence-associated genes, *ABA-related genes* and *MADS14*. This may be the reason for the larger-scale death of bamboo after flowering. This study suggests that removing the soil, killing the grass and fertilizing would help to develop sustainable management of *D. latiflorus*.

### 3.4. Protein Changes and Metabolism Relate to Floral Transition

Our iTRAQ data obtained from the L1 (vegetative state) and L2 (reproductive state) proteins facilitated a genome-scale proteomic analysis of bamboo floral transition. Here, a comparison of the L1 and L2 leaf proteomes in *D. latiflorus* revealed 721 proteins that are differentially expressed during floral transition. This proteomic change is on the same order of magnitude as changes in the genes at the transcriptional level, such as carbon metabolism, photosynthesis, and starch and sucrose metabolism pathways. However, a direct comparison of proteins and their corresponding mRNA expression levels revealed poor correlations and few significant overlapping changes. This finding is in agreement with previous studies on various organisms showing a large number of genes with inconsistency between the transcript protein levels, the Pearson correlation coefficients for these data range from 0.46 to 0.76 [39,40] and a substantial regulatory process occurs after mRNA (post-transcriptional, translational and protein degradation regulation) [18]. The relationship between mRNA and protein abundances is complex due to the series of involved regulatory processes. Schwanhausser et al. showed that mRNA is produced at a much slower rate than the rate of protein translation and that the protein products were five times more stable and 2800 times more abundant than the mRNAs in mammalian cells [41]. Therefore, the expression changes detected at the mRNA level may or may not result in variable protein abundance.

### 3.5. Proteome and Transcriptome Correlation Analysis

In our study, there were 55 proteins and transcripts showing the same trends. We found that Unigene129955 has a high consistency with the corresponding transcript [42]. The amino sugar and nucleotide sugar-related protein and photosynthesis-related protein corresponding to the transcript showed a decrease in parallel during floral transition, and NADP-ME (unigene9416) was upregulated in L2, which might even be in the position to influence Chlorophyll content and photosystem II activity. Therefore, the decreased functions related to stress and photosynthesis deserve further investigation as potentially significant regulators of death after large-scale bamboo flowering, which is in accordance with the RNA-seq results. PNI288, which is one subfamily of the bark storage protein (BSP) family, plays an overlapping but non-redundant role in N storage [43]. The key processes of this N redistribution are autumnal leaf senescence and the storage of released N as bark storage proteins (BSP) in perennial tissues.

**Figure 7:**
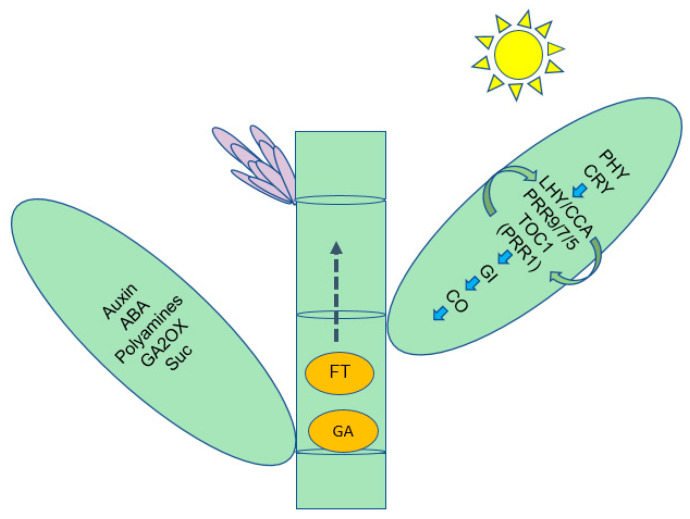
Summary of some of the biological pathways involved in *D. latiflorus* floral transition.

We demonstrated that bamboo flowering was the result of an orderly alteration of a series of physiological and biochemical events, such as sugar and acid metabolism, and that each of these metabolic systems or processes is involved in the regulation of some genes. In our present study, we proposed a pathway model depending on circadian rhythms to induce flowering in bamboo (Figure 7). This model involves metabolites, hormones and gene products interacting as long- or short-distance signaling molecules, which will lay the foundation for uncovering bamboo flowering.

## 4. Materials and Methods

### 4.1. Sample Preparation

*D. latiflorus* leaves were collected from each of the three flowering clumps and three vegetative clumps in 2011 near Longsuo village, Pengpu town, Mile county of Yunnan Province in southwest China (24°02′13″ N, 103°22′26″ E). All collected leaves were classified into four phases by their developmental processes. Three vegetative and reproductive clumps of similar sizes and developmental stages were selected. At the second to third leaf position from the branch’s top (in September, after the leaves stopped growing), the three whole fully extended leaves were harvested. We collected bamboo seeds to germinate and tested the different ages of the bamboo seedlings (Figure S2). Samples were immediately frozen in liquid nitrogen and stored at −80 °C until the RNA and/or protein was extracted, and the other leaves stored in FAA (formalin:glacial acetic:50% ethanol = 1:1:18) were used for morphological analysis.

### 4.2. Measurement of Leaf Surface Area

The leaf area was measured using the paper-cutting method described by Hattersley [44] with some modifications. Fresh leaves were collected and immediately processed for leaf area measurements. One piece of A3 size paper was taken and its weight and area were measured. The leaf was then placed on the paper and its outline was drawn carefully. The paper within the outlined area was then cut and weighed. The leaf area in cm^2^ was calculated according to the following formula:

(1) $$\mathrm{Leaf}\;\mathrm{area}\;=\;\frac{a\times y}{x}$$

where *a* = area of the A3 paper in cm^2^, *y* = the weight of the cut paper in g, and *x* = the weight of the A3 paper in g [45].

### 4.3. Leaf Anatomy, Leaf Stomatal Size and Density Measurement

For microscopic studies, leaf materials (4 × 4 mm) were cut next to the major first order veins at 50% of the whole leaf length. To examine the paraffin sectioning, the samples were fixed in FAA and dehydrated in a graded ethanol series. The samples were then embedded in Paraplast by LEICA EG 1160 (Leica Micosystems, Wetzlar, Germany). The Paraplast was cut into 5 μm sections on a Leica RM2135 microtome and mounted on glass slides. The sections were then deparaffinized with xylene, stained with fast green and analyzed using a Leica DFC 295 camera (Vashaw Scientific Inc., Wetzlar, Germany) attached to a Leica DM 1000. The rest were processed for SEM as previously described [46]. The lower epidermises of the leaves were coated in gold-palladium and analyzed by a Hitachi S-4800 scanning electron microscope (Hitachi High-Technologies Corp., Tokyo, Japan). The leaf stomatal size and density were analyzed by the Image J software [47].

### 4.4. Chlorophyll Fluorescence Detection in the D. latiflorus

The chlorophyll content of the four stages of leaves was determined using an Imaging-PAM chlorophyll fluorometer (Walz, Effeltrich, Germany). The leaves were placed in a dark room for 20 min. The instrument was driven by software that allows the user to control the timing, duration and intensity of each light source (LED measuring light panels, continuous blue actinic light and blue saturating light pulses) [48]. The kinetic images of the chlorophyll fluorescence depended on continuous modulated light. To analyze the heterogeneity of the chlorophyll fluorescence of leaves, at least 3 points were randomly selected on the surface of the leaves. Each pixel value among these three points and fluorescence parameters was automatically calculated [49] using the Imaging Win software. All parameter measurements were repeated at least three times.

### 4.5. RNA-seq, Data Processing and Reference Preparation

The total RNA of leaves in the four stages (L0, L1, L2 and L3) was isolated using RNAiso Plus (TaKaRa, Shiga, Japan). Equal amounts of RNA collected from three independent experiments were used for sequencing. The-RNA-seq libraries were constructed with the methods previously described [50]. Finally, the pooled library was sequenced using an Illumina HiSeq^TM^ 2000. High-quality reads (clean reads) were obtained by removing low-quality reads with ambiguous nucleotides, and adaptor sequences were filtered from the raw reads. The de novo assembly for each of these 12 datasets (leaves of the four stages of *D. latiflorus*, three biological repeats) was performed separately by Trinity [51]. As there was no reference genome available for *D. latiflorus* (the generated unigenes (hereafter referred to as dataset 1)), our previous obtained transcriptomes of the floral buds [50] ((referred to as dataset 2), roots, leafs and seeds of *D. latiflorus* [52] (dataset 3)) as well as the *D. latiflorus* EST data (release 20121230, referred to as dataset 4) were assembled into a reference by TGICL and used to remove redundancy by CD-HIT-EST [53].

### 4.6. Screening of DEGs

RSEM (v1.1.11) [54] was used to quantify the transcript abundance in each sample, and then the RSEM-estimated fragment counts were fed into the edgeR package [55]. Gene expression bias was evaluated using Fisher’s exact test in the edger package. We followed the methods in [15] for screening the key candidate genes that indicate the vegetative to the reproductive phase change. The phase change from L1 to L3 showed the floral transition in the same culms of flowering clumpling (sympodial). These genes were clustered using k-means, with k = 100 and 1000 repeats of the “k-means” function in the Gene cluster 3.0 [56] and Java Treeview software [57]. The hierarchical tree was constructed using the function “gplots” from the R package “ggplot” [58].

To deduce their putative functions, the differentially expressed genes were subjected to BLASTX analysis (e-value: 1 × 10^−5^) against the UniprotKB and NR database. The BLAST2GO program [59] was used to perform the gene ontology (GO) [60] annotation. We performed a cluster analysis of the gene expression patterns with the cluster [56] software and the Java Treeview software [57].

Sequences were annotated by a homology search against NCBI and aligned via Clustal W. The phylogenetic trees of the homologous genes were constructed by employing the neighbor-joining method of MEGA5.05 [61] with 1000 bootstrap replicates.

### 4.7. qRT-PCR Validation

Twenty pairs of primers were designed to generate the amplicons for validating the RNA-seq data (Table S3). Gene-specific primers were designed using Primer Express 3.0. Aliquots of total RNA extracted for sequencing, as described earlier, were used for qRT-PCR experiments according to the manufacturer’s instructions (Roche, Shanghai, China). The *EF1α* gene was used as a reference in all the qRT-PCR experiments [62].

### 4.8. iTRAQ Labeling and SCX Fractionation

Samples for protein extraction were prepared with two biological replications. The total protein (100 μg) was taken from each sample solution and then the protein was digested with Trypsin Gold (Promega, Madison, WI, USA) with a ratio of protein:trypsin = 30:1 at 37 °C for 16 h. After trypsin digestion, the peptides were dried by vacuum centrifugation. The peptides were reconstituted in 0.5 M TEAB and processed according to the manufacture’s protocol for the 8-plex iTRAQ reagent (Applied Biosystems, Waltham, MA, USA).

SCX chromatography was performed with a LC-20AB HPLC Pump system (Shimadzu, Kyoto, Japan). The iTRAQ-labeled peptide mixtures were reconstituted with 4 mL buffer A (25 mM NaH_2_PO_4_ in 25% ACN, pH 2.7) and loaded onto a 4.6 × 250 mm Ultremex SCX column containing 5-μm particles (Phenomenex, Torrance, CA, USA). The peptides were eluted at a flow rate of 1 mL/min with a gradient of buffer A for 10 min, 5–60% buffer B (25 mM NaH_2_PO_4_, 1 M KCl in 25% ACN, pH 2.7) for 27 min, and 60–100% buffer B for 1 min. The system was then maintained at 100% buffer B for 1 min before equilibrating it with buffer A for 10 min prior to the next injection. The elution was monitored by measuring the absorbance at 214 nm, and fractions were collected every 1 min. The eluted peptides were pooled into 20 fractions, desalted with a Strata X C18 column (Phenomenex) and vacuum-dried.

### 4.9. LC-ESI-MS/MS Analysis Based on Q EXACTIVE

Each fraction was resuspended in buffer A (2% ACN, 0.1%FA) and centrifuged at 20,000× *g* for 10 min; the final concentration of the peptide was about 0.5 μg/μL on average. In total, 10 μL supernatant on a LC-20AD nanoHPLC (Shimadzu, Kyoto, Japan) was loaded by an autosampler onto a 2 cm C18 trap column. Then, the peptides were eluted onto a 10 cm analytical C18 column (inner diameter 75 μm) packed in-house. The samples were loaded at 8 μL/min for 4 min, and then the 44 min gradient was run at 300 nL/min starting from 2 to 35% B (98% ACN, 0.1%FA), followed by a 2 min linear gradient to 80%, and maintenance at 80% B for 4 min, and finally a return to 5% for 1 min.

The peptides were subjected to nanoelectrospray ionization followed by tandem mass spectrometry (MS/MS) in a Q EXACTIVE (Thermo Fisher Scientific, San Jose, CA, USA) device coupled online to the HPLC. Intact peptides were detected in the Orbitrap at a resolution of 70,000. Peptides were selected for MS/MS using the high-energy collision dissociation (HCD) operating mode with a normalized collision energy setting of 27.0; ion fragments were detected in the Orbitrap at a resolution of 17,500. The electrospray voltage applied was 1.6 kV. Automatic gain control (AGC) was used to optimize the spectra generated by the orbitrap. The AGC target for full MS was 3e6, and 1e5 was used for MS2. For MS scans, the m/z scan range was 350 to 2000 Da. For MS2 scans, the *m*/*z* scan range was 100–1800.

### 4.10. Data Analysis and Functional Annotation

Raw data files acquired from the Orbitrap were converted into MGF files using Proteome Discoverer software (ver. 2.2, Thermo Fisher Scientific, Waltham, MA, USA). Protein identification was analyzed by a thorough search using the Mascot software (Matrix Science, London, UK; version 2.3.02) against NCBI Poaceae (456,311 sequences). Mascot was queried with a fragment ion mass tolerance of 0.050 Da, a parent ion tolerance of 20.0 ppm, and allowance for one missed cleavage in the trypsin digests. To reduce the probability of false peptide identification, only peptides with significance scores (≥20) at a 99% confidence interval greater than “identity” determined using a Mascot probability analysis were counted as identified. Each positive protein identification contained at least one unique peptide. Proteins containing at least two unique spectra were selected for quantification analysis. The quantitative protein ratios were weighted and normalized by the median ratio in Mascot. We only used ratios with *p*-values < 0.05, and only fold changes of >1.2 was considered significant. Functional annotations of the proteins were conducted using the Blast2GO program against the non-redundant protein database (NR; NCBI). The KEGG database (http://www.genome.jp/kegg/) and the COG database (http://www.ncbi.nlm.nih.gov/COG/) were used to classify and group these identified proteins.

### 4.11. Availability of Sequence Data

The Illumina reads and the mass spectrometry proteomics data generated in this study have been deposited in Genome Sequence Archive (https://bigd.big.ac.cn/gsa/). The BioProjects are PRJCA003711 and PRJCA003741, respectively. In addition, the raw data in this study can be downloaded directly in http://www.genobank.org/Data/Dlatiflorus.html.

## 5. Conclusions

Woody bamboos have unique flowering habits, but little information is available on the molecular mechanisms of bamboo flowering. In this study, we presented the application of RNA-seq to study leaves and reported a comprehensive analysis of the transcriptome, which will serve as a blueprint for the gene expression profile during floral transition. Many genes and proteins associated with floral transition were detected, including flowering biomarker genes. And we found that the photoperiod pathways may play an essential role in bamboo’s floral transition. In addition, combined with transcriptomic and proteomic data, we identified biological processes related to bamboo flowering, such as carbon metabolism as well as starch and sucrose metabolism. Finally, we proposed a pathway model of bamboo floral transition. In this way, the integrated analysis enabled a comprehensive understanding of the biological events relevant to the regulation of bamboo floral transition, and our data will provide new insights into the molecular mechanism of the bamboo flowering regulatory network.

## Figures and Tables

**Table 1:** Summary for the outcome of the reference sequences using the four datasets in *D. latiflorus.*

Summary	Number
Total number of unigenes	155,494
Total length	229,730,264
Mean unigene length	1477
N50 (bp)	2069
N90 (bp)	779

**Table 2:** The DEGs of major floral pathway loci and genes between vegetative leaves and flowering leaves (L0 vs L3).

Gene ID	Gene Name	Predicted Protein	Pathway	Log2FC
Unigene2563	*FT*	Putative kinase inhibitor	Pathway integrator	−2.81
Unigene123271	*SOC1*	MADS-box	Pathway integrator	−8.71
Unigene39652	*AGL24*	MADS-box	Pathway integrator	7.37
Unigene57034	*Fri*	Coiled-coil domain	Vernalization	−6.04
Unigene110221	*VIN3*	PHD, VID-domain	Vernalization	6.69
Unigene120387	*FCA*	RNA-binding	Autonomous	10.74
Unigene132444	*FY*	Polyadenylation factor	Autonomous	2.64
Unigene132606	*GID1*	Gibberellin receptor GID1	Gibberellin	8.79
Unigene33012	*GA20ox*	Gibberellin 20 oxidase 1-B	Gibberellin	−6.88
Unigene29825	*SPL9*	Squamosa promoter-binding-like protein 9	Age pathway	−6.11
Unigene30392	*MADS14*	MADS-box	Meristem identity, Floral organ identity	−8.11
Unigene108692	*AGL6*	MADS-box	Other flowering gene	10.02
Unigene72731	*EMF1*	Polycomb-group (Pc-G) proteins	Other flowering gene	7.43

**Table 3:** The main differential genes of circadian rhythms that regulate flowering during photoperiod between L0 and L3.

Gene ID	Gene Annotation	FPKM		Log2FC
		L0	L3	
Unigene42305	Phytochrome A (Phy A)	0.01	1.09	6.77
Unigene28224	Phytochrome B (Phy B)	0.21	4.23	4.33
Unigene81404	Cryptochrome (Cry)	9.01	54.54	2.60
Unigene10798	Gigantea (GI)	0.42	5.57	3.73
Unigene118524	Two-component response regulator-like APRR5 (PRR5)	0.01	14.92	10.54
Unigene4579	Two-component response regulator-like APRR7 (PRR7)	5.65	0.01	−9.14
Unigene105685	Two-component response regulator-like APRR9 (PRR9)	0.01	9.9	9.95
Unigene91042	Two-component response regulator-like APRR9 (PRR3)	3.98	0.01	−8.64
Unigene105693	Two-component response regulator-like APRR1 (PRR1/TOC1)	83.05	21.21	−1.97
Unigene99793	Homeobox-leucine zipper protein HDG6 (FWA)	0.82	4.4	2.42
Unigene2906	protein LHY	0.01	4.02	8.65
Unigene7391	EARLY FLOWERING 3 (ELF3)	0.01	2.13	7.73
Unigene24623	EARLY FLOWERING 4 (ELF4)	0.98	0.01	−6.61
Unigene5825	Protein CCA1	0.69	4.78	2.79
Unigene56852	COP1	13.94	2.17	−2.68
Unigene102233	CRY	4.46	0.82	−2.44
Unigene26943	PIF3	2.41	0.01	−7.91
Unigene26128	SPA1	0.06	2.59	5.43
Unigene75776	PAP1	0.58	15.43	4.73
Unigene148518	CHS	13.97	121.68	3.12
Unigene56158	CK2β	0.24	3.56	3.89
Unigene129590	HY5	1.45	15.35	3.40

**Table 4:** Correlation analysis of the differentially expressed proteins and differentially expressed genes.

Unigene ID	Protein ID	Proteins log2FC	Transcripts log2FC	Description
Unigene36630	P1053	0.41	1.51	*psbP*; photosystem II oxygen-evolving enhancer protein 2
Unigene124766	P3937	−1.33	−0.23	PsbS protein
Unigene62245	P536	−0.77	−2.94	*psaH*; photosystem I subunit VI
Unigene9416	P3559	1.00	8.83	NADP dependent malic enzyme
Unigene117107	P1873	−0.30	−0.45	*glgC*; glucose-1-phosphate adenylyltransferase
Unigene148716	P639	−3.27	−2.47	Chitinase
Unigene111502	P1156	−1.81	−0.16	Armadillo-like
Unigene129955	P977	−1.10	−1.15	vegetative storage protein PNI288

## Supplementary Materials

The following are available online at https://www.mdpi.com/1422-0067/21/22/8430/s1, Figure S1: The length distribution of reconstructed reference sequences (dataset 1, dataset 2, dataset 3 and dataset 4) in *D. latiflorus*. Figure S2: Quantitative RT-PCR validations. Figure S3: These unigenes were assigned into different pathways. Figure S4: Senescence-associated genes during floral transition in *D. latiflorus*. Figure S5: Plant hormone signal transduction for unigenes by KEGG annotation. Figure S6: The bamboo seedlings of different ages. Figure S7: COG functional classifications of the *D. latiflorus* proteomics. Figure S8: Concordance between changes in the protein and corresponding transcripts. Table S1: Summary of sequencing data and alignment statistics. Table S2: Statistics of annotation results for *D. latiflorus* unigenes. Table S3: Primers pairs used for Quantitative Real-Time PCR. Table S4: Expression of the same clump of *D. latiflorus* flowering biomarkers in floral transition. Table S5: The pathway and the products involved in the pathway of plant hormone signal transduction. Table S6: key unigenes sequences. Table S7: 4629 protein sequences and annotation information. Table S8: 282 up-regulated in the differentially expressed proteins. Table S9: 439 down-regulated in differentially expressed protein.

## Abbreviations

RNA-seq	High-throughput RNA sequencing
KEGG	Kyoto Encyclopedia of Genes and Genomes
SAM	Shoot apical meristem
ABA	Abscisic acid
iTRAQ	Isobaric tags for relative and absolute quantitation
ETR	Electron transfer reactions
PAR	Photosynthetic active radiation
GO	Gene ontology
Nr	Non-redundant protein database
EC	Enzyme Commission
Fri	Frigida
PRR5	Response regulator-like APRR5
GAs	Gibberellins
GAI	Gibberellin insensitive
GI	Gigantea
CO	Constans
TOC1	Timing of cab expression 1
LHY	Late elongated hypocotyl
CCA1	Circadian clock associated 1
qRT-PCR	Quantitative real-time PCR
NADP-ME	NADP dependent malic enzyme
BSP	Bark Storage Proteins
DEGs	Differentially expressed genes
HCD	High-energy collision dissociation
AGC	Automatic gain control

## References

[1] Poethig R.S. (1990). Phase Change and the Regulation of Shoot Morphogenesis in Plants. Science.

[2] Cho L.-H., Yoon J., An G. (2017). The control of flowering time by environmental factors. Plant J..

[3] Shrestha R., Gómez-Ariza J., Brambilla V., Fornara F. (2014). Molecular control of seasonal flowering in rice, arabidopsis and temperate cereals. Ann. Bot..

[4] Fornara F., De Montaigu A., Coupland G. (2010). SnapShot: Control of Flowering in Arabidopsis. Cell.

[5] Komiya R., Ikegami A., Tamaki S., Yokoi S., Shimamoto K. (2008). Hd3a and RFT1 are essential for flowering in rice. Development.

[6] Brandis D. (1899). Biological Notes on Indian Bamboos.

[7] Böhlenius H., Huang T., Charbonnel-Campaa L., Brunner A.M., Jansson S., Strauss S.H., Nilsson O. (2006). CO/FT Regulatory Module Controls Timing of Flowering and Seasonal Growth Cessation in Trees. Science.

[8] Keeley J.E., Bond W.J. (1999). Mast Flowering and Semelparity in Bamboos: The Bamboo Fire Cycle Hypothesis. Am. Nat..

[9] Janzen D.H. (1976). Why Bamboos Wait So Long to Flower. Annu. Rev. Ecol. Syst..

[10] Tian Z., Liu X., Fan Z., Liu J., Pimm S.L., Liu L., Garcia C., Songer M., Shao X., Skidmore A. (2019). The next widespread bamboo flowering poses a massive risk to the giant panda. Biol. Conserv..

[11] Guo X., Wang Y., Wang Q., Xu Z., Lin X. (2015). Molecular characterization of FLOWERING LOCUS T(FT)genes from bamboo (*Phyllostachys violascens*). J. Plant Biochem. Biotechnol..

[12] Shih M.-C., Chou M.-L., Yue J.-J., Hsu C.-T., Chang W.-J., Ko S.-S., Liao D.-C., Huang Y.-T., Chen J.-W., Yuan J.-L. (2014). BeMADS1 is a key to delivery MADSs into nucleus in reproductive tissues-De novo characterization of *Bambusa edulis* transcriptome and study of MADS genes in bamboo floral development. BMC Plant Biol..

[13] Jiao Y., Hu Q., Zhu Y., Zhu L., Ma T., Zeng H., Zang Q., Li X., Lin X. (2019). Comparative transcriptomic analysis of the flower induction and development of the Lei bamboo (*Phyllostachys violascens*). BMC Bioinform..

[14] Gan C.S., Chong P.K., Pham T.K., Wright P.C. (2007). Technical, Experimental, and Biological Variations in Isobaric Tags for Relative and Absolute Quantitation (iTRAQ). J. Proteome Res..

[15] Wang X., Zhang X., Zhao L., Guo Z.-H. (2014). Morphology and Quantitative Monitoring of Gene Expression Patterns during Floral Induction and Early Flower Development in *Dendrocalamus latiflorus*. Int. J. Mol. Sci..

[16] Hartigan J.A., Wong M.A. (1979). Algorithm AS 136: A k-means clustering algorithm. JSTOR.

[17] Vogel C., Marcotte E.M. (2012). Insights into the regulation of protein abundance from proteomic and transcriptomic analyses. Nat. Rev. Genet..

[18] Johanson U. (2000). Molecular Analysis of FRIGIDA, a Major Determinant of Natural Variation in Arabidopsis Flowering Time. Science.

[19] Liu S.N., Zhu L.F., Lin X., Ma L. (2016). Overexpression of the repressor gene PvFRI-L from *Phyllostachys violascens* delays flowering time in transgenic *Arabidopsis thaliana*. Biol. Plant..

[20] Sakamoto T., Miura K., Itoh H., Tatsumi T., Ueguchi-Tanaka M., Ishiyama K., Kobayashi M., Agrawal G.K., Takeda S., Abe K. (2004). An Overview of Gibberellin Metabolism Enzyme Genes and Their Related Mutants in Rice. Plant Physiol..

[21] Murase K., Hirano Y., Sun T.-P., Hakoshima T. (2008). Gibberellin-induced DELLA recognition by the gibberellin receptor GID1. Nat. Cell Biol..

[22] Ohmori S., Kimizu M., Sugita M., Miyao A., Hirochika H., Uchida E., Nagato Y., Yoshida H. (2009). MOSAIC FLORAL ORGANS1, an AGL6-Like MADS Box Gene, Regulates Floral Organ Identity and Meristem Fate in Rice. Plant Cell.

[23] Sung Z.R., Belachew A., Shunong B., Bertrand-Garcia R. (1992). EMF, an Arabidopsis Gene Required for Vegetative Shoot Development. Science.

[24] Wu G., Park M.Y., Conway S.R., Wang J.-W., Weigel D., Poethig R.S. (2009). The Sequential Action of miR156 and miR172 Regulates Developmental Timing in Arabidopsis. Cell.

[25] Guo Z.-H., Ma P.-F., Yang G.-Q., Hu J.-Y., Liu Y.-L., Xia E.-H., Zhong M.-C., Zhao L., Sun G.-L., Xu Y.-X. (2019). Genome Sequences Provide Insights into the Reticulate Origin and Unique Traits of Woody Bamboos. Mol. Plant.

[26] Theissen G., Michaels S.D., Ditta G., Gustafson-Brown C., Pelaz S., Yanofsky M., Amasino R.M. (2003). Faculty Opinions recommendation of AGL24 acts as a promoter of flowering in Arabidopsis and is positively regulated by vernalization. Fac. Opin. Publ. Peer Rev. Biomed. Lit..

[27] Searle I., Coupland G. (2004). Induction of flowering by seasonal changes in photoperiod. EMBO J..

[28] Hayama R., Coupland G. (2003). Shedding light on the circadian clock and the photoperiodic control of flowering. Curr. Opin. Plant Biol..

[29] Alabadí D., Oyama T., Yanovsky M.J., Harmon F.G., Más P., Kay S.A. (2001). Reciprocal Regulation Between TOC1 and LHY/CCA1 Within the Arabidopsis Circadian Clock. Science.

[30] Dutta S., Biswas P., Chakraborty S., Mitra D., Pal A., Das M. (2018). Identification, characterization and gene expression analyses of important flowering genes related to photoperiodic pathway in bamboo. BMC Genom..

[31] Hisamoto Y., Kobayashi M. (2012). Flowering habit of two bamboo species, Phyllostachys meyeri and Shibataea chinensis, analyzed with flowering gene expression. Plant Species Biol..

[32] Kobayashi Y., Weigel D. (2007). Move on up, it’s time for change—Mobile signals controlling photoperiod-dependent flowering. Genes Dev..

[33] Samach A., Onouchi H., Gold S.E., Ditta G.S., Schwarz-Sommer Z., Yanofsky M.F., Coupland G. (2000). Distinct Roles of CONSTANS Target Genes in Reproductive Development of Arabidopsis. Science.

[34] Bernier G., Périlleux C. (2005). A physiological overview of the genetics of flowering time control. Plant Biotechnol. J..

[35] Flexas J., Badger M., Chow W.S., Medrano H., Osmond C.B. (1999). Analysis of the Relative Increase in Photosynthetic O2 Uptake When Photosynthesis in Grapevine Leaves Is Inhibited following Low Night Temperatures and/or Water Stress. Plant Physiol..

[36] McWilliam J., Kramer P., Musser R. (1982). Temperature-Induced Water Stress in Chilling-Sensitive Plants. Funct. Plant Biol..

[37] Song S., Qi T., Fan M., Zhang X., Gao H., Huang H., Wu D., Guo H., Xie D. (2013). The bHLH Subgroup IIId Factors Negatively Regulate Jasmonate-Mediated Plant Defense and Development. PLoS Genet..

[38] Kobayashi K., Yasuno N., Sato Y., Yoda M., Yamazaki R., Kimizu M., Yoshida H., Nagamura Y., Kyozuka J. (2012). Inflorescence meristem identity in rice is specified by overlapping functions of three AP1/FUL-like MADS box genes and PAP2, a SEPALLATA MADS box gene. Plant Cell.

[39] Gallardo K., Firnhaber C., Zuber H., Héricher D., Belghazi M., Henry C., Küster H., Thompson R. (2007). A Combined Proteome and Transcriptome Analysis of Developing Medicago truncatula Seeds. Mol. Cell. Proteom..

[40] Griffin T.J., Gygi S.P., Ideker T., Rist B., Eng J., Hood L., Aebersold R. (2002). Complementary Profiling of Gene Expression at the Transcriptome and Proteome Levels in *Saccharomyces cerevisiae*. Mol. Cell. Proteom..

[41] Schwanhäusser B., Busse D., Li N., Dittmar G., Schuchhardt J., Wolf J., Chen W., Selbach M. (2011). Global quantification of mammalian gene expression control. Nat. Cell Biol..

[42] Wildhagen H., Dürr J., Ehlting B., Rennenberg H. (2010). Seasonal nitrogen cycling in the bark of field-grown Grey poplar is correlated with meteorological factors and gene expression of bark storage proteins. Tree Physiol..

[43] Cooke J.E.K., Weih M. (2005). Nitrogen storage and seasonal nitrogen cycling in Populus: Bridging molecular physiology and ecophysiology. New Phytol..

[44] Hattersley P.W. (1984). Characterization of C4 Type Leaf Anatomy in Grasses (Poaceae). Mesophyll: Bundle Sheath Area Ratios. Ann. Bot..

[45] Minaxi, Saxena J. (2010). Disease suppression and crop improvement in moong beans (*Vigna radiata*) through Pseudomonas and Burkholderia strains isolated from semi arid region of Rajasthan, India. BioControl.

[46] Marrie T.J., Nelligan J., Costerton J.W. (1982). A scanning and transmission electron microscopic study of an infected endocardial pacemaker lead. Circulation.

[47] Abràmoff M.D., Magalhães P.J., Ram S.J. (2004). Image processing with ImageJ. Biophotonics Int..

[48] Nedbal L., Soukupová J., Kaftan D., Whitmarsh J., Trtílek M. (2000). Kinetic imaging of chlorophyll fluorescence using modulated light. Photosynth. Res..

[49] Genty B., Briantais J.-M., Baker N.R. (1989). The relationship between the quantum yield of photosynthetic electron transport and quenching of chlorophyll fluorescence. Biochim. Biophys. Acta (BBA) Gen. Subj..

[50] Zhang X.-M., Zhao L., Larson-Rabin Z., Li D.-Z., Guo Z.-H. (2012). De Novo Sequencing and Characterization of the Floral Transcriptome of *Dendrocalamus latiflorus* (Poaceae: Bambusoideae). PLoS ONE.

[51] Grabherr M.G., Haas B.J., Yassour M., Levin J.Z., Thompson D.A., Amit I., Adiconis X., Fan L., Raychowdhury R., Zeng Q. (2011). Full-length transcriptome assembly from RNA-Seq data without a reference genome. Nat. Biotechnol..

[52] Liu M., Qiao G., Jiang J., Yang H., Xie L., Xie J., Zhuo R. (2012). Transcriptome Sequencing and De Novo Analysis for Ma Bamboo (*Dendrocalamus latiflorus* Munro) Using the Illumina Platform. PLoS ONE.

[53] Li W., Godzik A. (2006). Cd-hit: A fast program for clustering and comparing large sets of protein or nucleotide sequences. Bioinformatics.

[54] Li B., Dewey C.N. (2011). RSEM: Accurate transcript quantification from RNA-Seq data with or without a reference genome. BMC Bioinform..

[55] Robinson M.D., McCarthy D.J., Smyth G.K. (2010). edgeR: A Bioconductor package for differential expression analysis of digital gene expression data. Bioinformatics.

[56] De Hoon M., Imoto S., Nolan J., Miyano S. (2004). Open source clustering software. Bioinformatics.

[57] Saldanha A.J. (2004). Java Treeview—Extensible visualization of microarray data. Bioinformatics.

[58] Wickham H., Chang W. ggplot2: An Implementation of the Grammar of Graphics. R Package Version 07. http://cranR-projectorg/package=ggplot2.

[59] Conesa A., Götz S., García-Gómez J.M., Terol J., Talón M., Robles M. (2005). Blast2GO: A universal tool for annotation, visualization and analysis in functional genomics research. Bioinformatics.

[60] Tatusov R.L., Fedorova N.D., Jackson J.D., Jacobs A.R., Kiryutin B., Koonin E.V., Krylov D.M., Mazumder R., Mekhedov S.L., Nikolskaya A.N. (2003). The COG database: An updated version includes eukaryotes. BMC Bioinform..

[61] Tamura K., Peterson N., Stecher G., Nei M., Kumar S. (2011). MEGA5: Molecular Evolutionary Genetics Analysis Using Maximum Likelihood, Evolutionary Distance, and Maximum Parsimony Methods. Mol. Biol. Evol..

[62] Liu M., Jiang J., Han X., Qiao G., Zhuo R. (2014). Validation of Reference Genes Aiming Accurate Normalization of qRT-PCR Data in *Dendrocalamus latiflorus* Munro. PLoS ONE.

