# Supplementary material for: Complementary Transcriptome and Proteome Analyses Provide Insight into the Floral Transition in Bamboo (Dendrocalamus latiflorus Munro)
Source: Int J Mol Sci. 2020 Nov 10;21(22):8430. doi: 10.3390/ijms21228430 (PMC7696756; doi:10.3390/ijms21228430)
Supplement: Supplementary file 1 [file ijms-21-08430-s001.zip › SUPPL/Supplementary Materials.pdf]

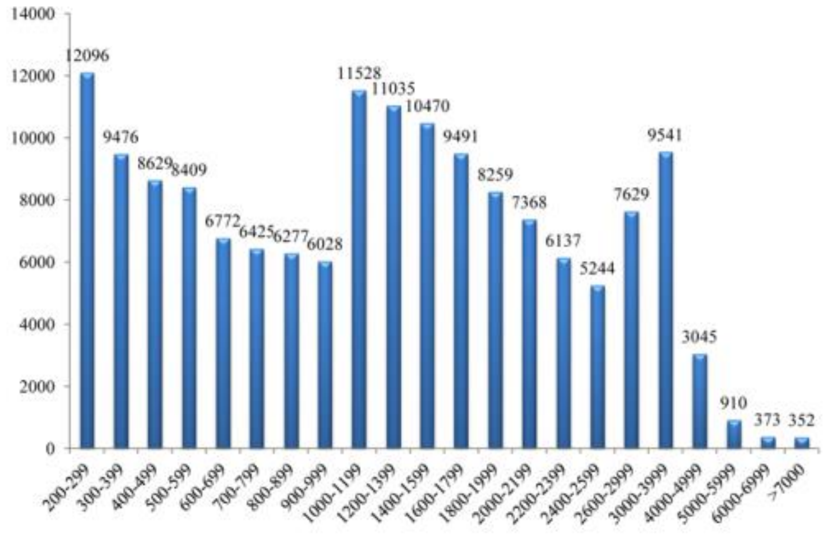

Figure S1. The length distribution of reconstructed reference sequences (dataset 1, dataset 2, dataset 3 and dataset 4) in *D. latiflorus.*

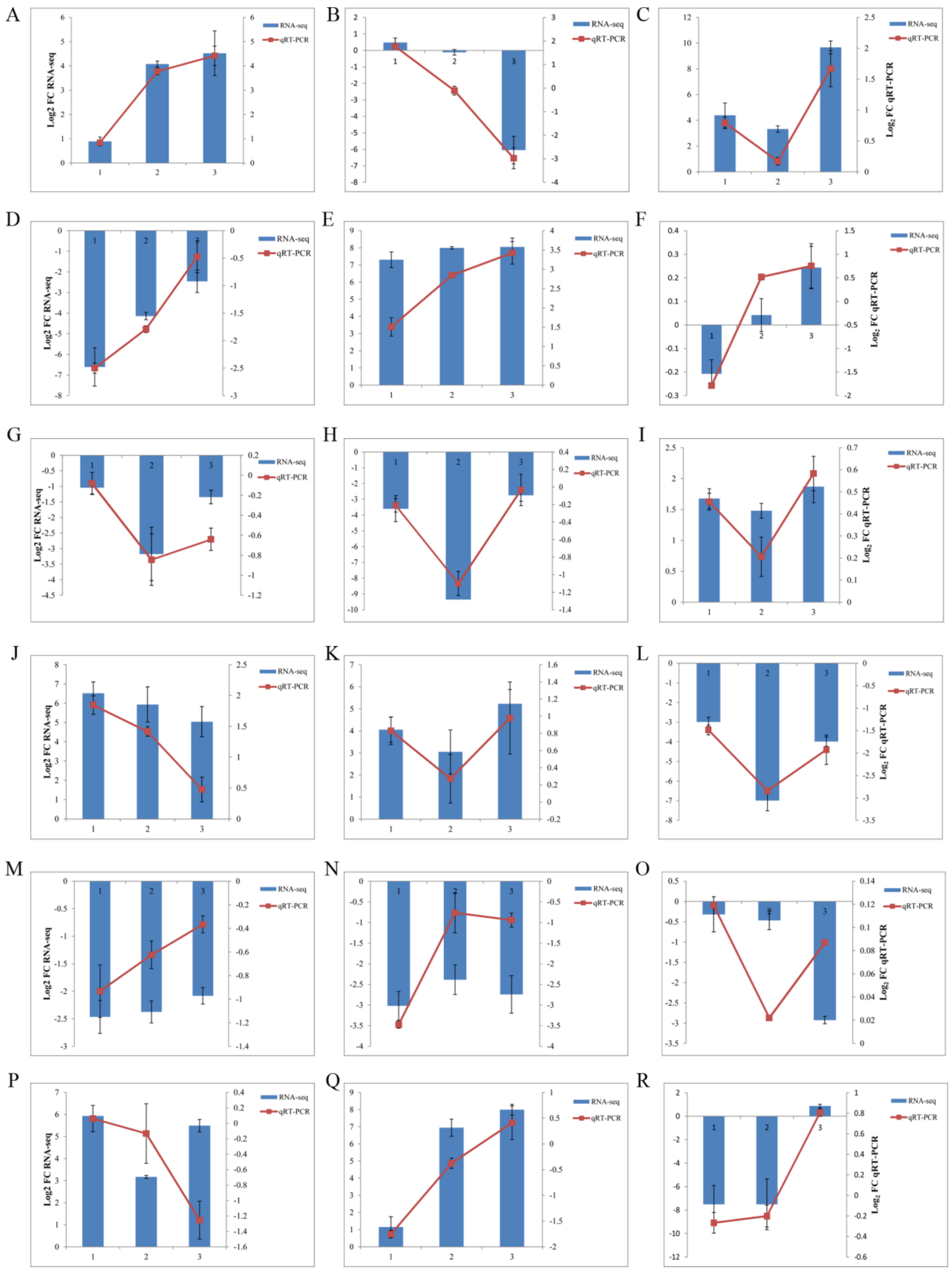

Figure S2. Quantitative RT-PCR validations**.** 18 genes were selected for the quantitative RT-PCR analysis, including Unigene49490 (**A**), Unigene57034 (B), Unigene108692 (C), Unigene32545 (**D**), Unigene114564 (E), Unigene120387 (F), Unigene103892 (G), Unigene104205 (H), Unigene109097 (I), Unigene110315 (J), Unigene130058 (K), Unigene137345 (L), Unigene30387 (M), Unigene32039 (N), Unigene33763 (O)**,** Unigene36923 (P), Unigene39652 (Q), Unigene28224 (R) in L0, L1, L2 and L3. 1, 2, 3 showed the relative expression level of L1, L2, L3 compared to L0. Vertical lines represent standard error for an average of three biological replicates.

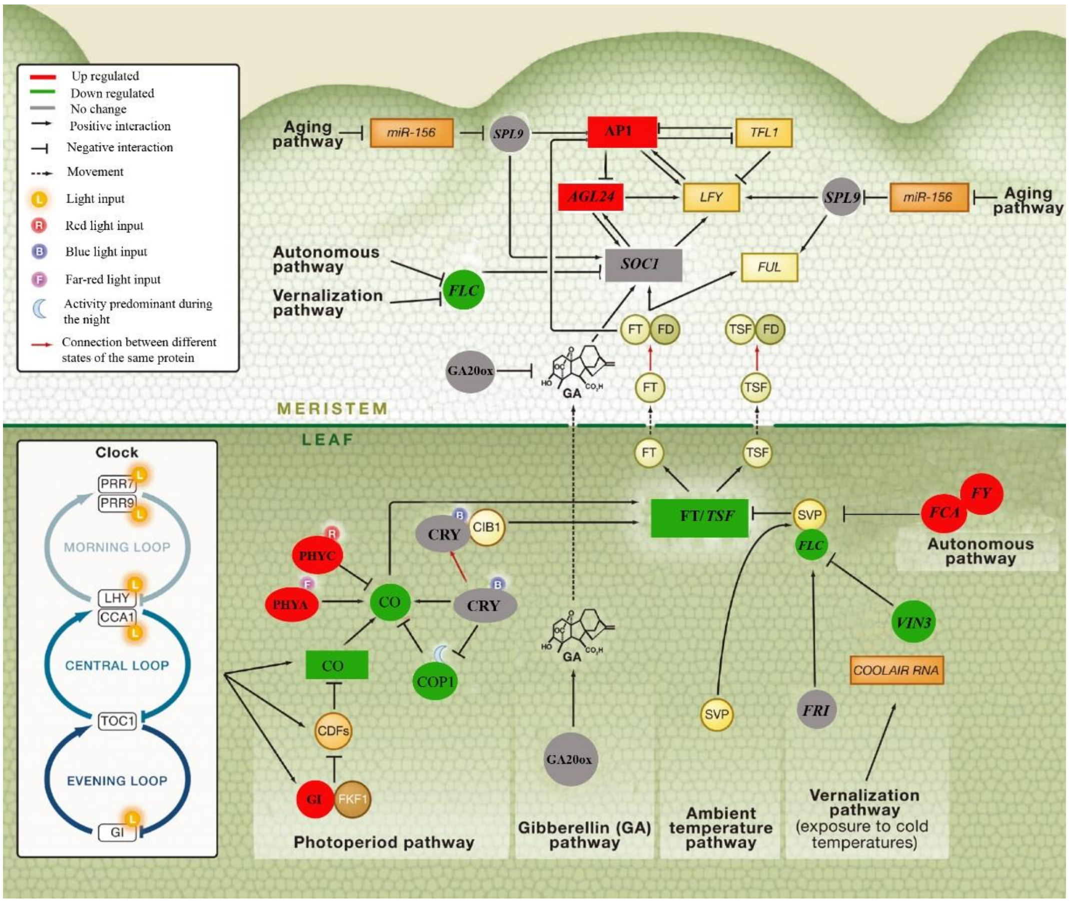

Figure S3. These unigenes were assigned into different pathways**.** Red indicated up-regulated, green indicated down-regulated and gray indicated no change in gene expression level. The gene expression level displayed L3 compared to the expression level of L0(Fornara, de Montaigu, & Coupland, 2010).

A

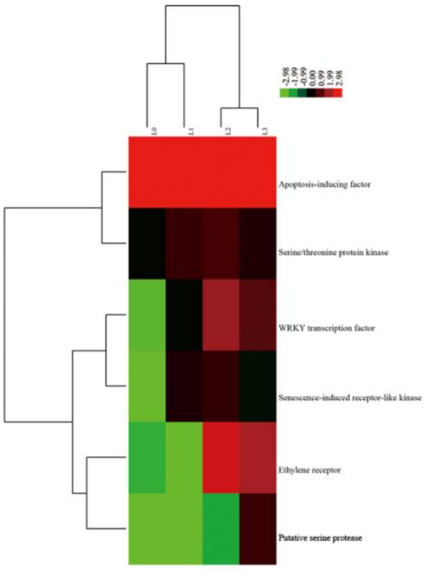

B

<table>
<tr><th rowspan="2">Gene ID</th><th rowspan="2">Functional description</th><th rowspan="2">Accession</th><th rowspan="2">E-value</th><th colspan="4">FPKM</th></tr>
<tr><th>L0</th><th>L1</th><th>L2</th><th>L3</th></tr>
<tr><td>Unigene108536</td><td>Putative serine protease</td><td>sp|P90893</td><td>6e-29</td><td>0.01</td><td>0.04</td><td>0.24</td><td>1.62</td></tr>
<tr><td>Unigene118824</td><td>Serine/threonine protein kinase</td><td>sp|P13185</td><td>1e-40</td><td>0.92</td><td>1.59</td><td>1.8</td><td>1.31</td></tr>
<tr><td>Unigene25575</td><td>Apoptosis-inducing factor</td><td>sp|Q96NN9</td><td>5e-26</td><td>30.27</td><td>40.86</td><td>52.33</td><td>148.67</td></tr>
<tr><td>Unigene26011</td><td>WRKY transcription factor</td><td>sp|Q9SZ67</td><td>1e-06</td><td>0.15</td><td>0.93</td><td>3.69</td><td>2.06</td></tr>
<tr><td>Unigene28224</td><td>Ethylene receptor</td><td>sp|Q9ZWL6</td><td>2e-20</td><td>0.21</td><td>0.01</td><td>6.13</td><td>4.23</td></tr>
<tr><td>Unigene28507</td><td>Senescence-induced receptor-like kinase</td><td>sp|O64483</td><td>7e-43</td><td>0.01</td><td>1.28</td><td>1.53</td><td>0.88</td></tr>
</table>

Figure S4. Senescence-associated genes during floral transition in *D. latiflorus*. A. Heat map showing relative expression of senescence-associated genes in the L0, L1, L2 and L3. Each row represents a gene. Expression differences are shown in different colors. Red means high expression and green means low expression. B. Tabular summary of expression values used to generate the heat map, with high FPKM value combinations highlighted in gray.

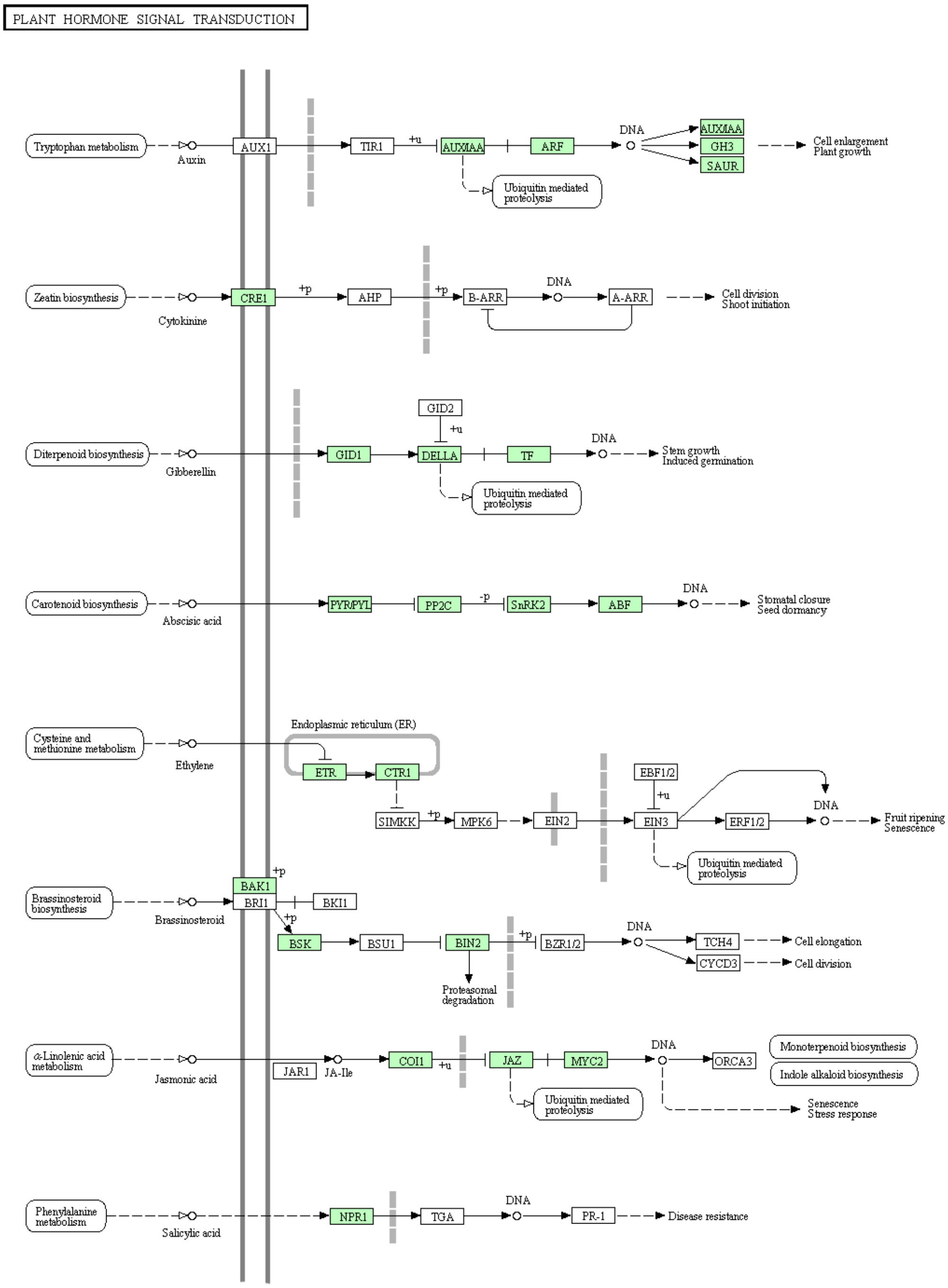

Figure S5. Plant hormone signal transduction for unigenes by KEGG annotation.

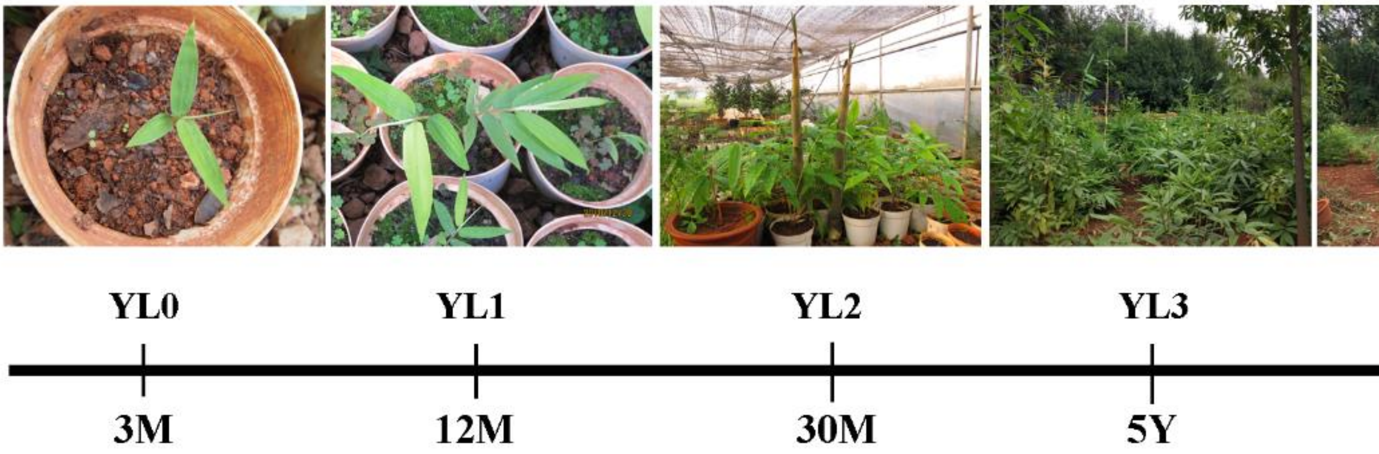

Figure S6. The bamboo seedlings of different ages**.** YL0: the seedlings of 3 months; YL1: the seedlings of 12 months; YL2: the seedlings of 30 months; YL3: the seedlings of 5 years; YL4: the seedlings of 6 years. YL0, YL1 and YL2 were planted in the greenhouse. YL3 and YL4 were planted out of the greenhouse. .

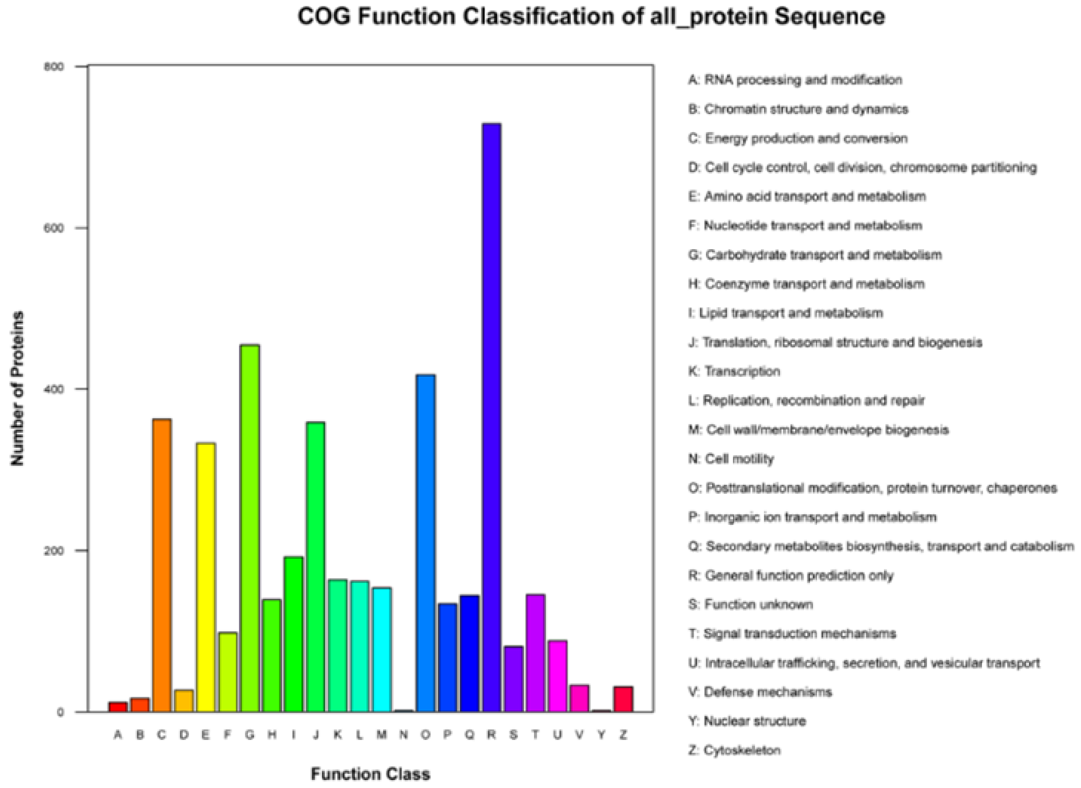

Figure S7. COG functional classifications of the D. latiflorus proteomics.

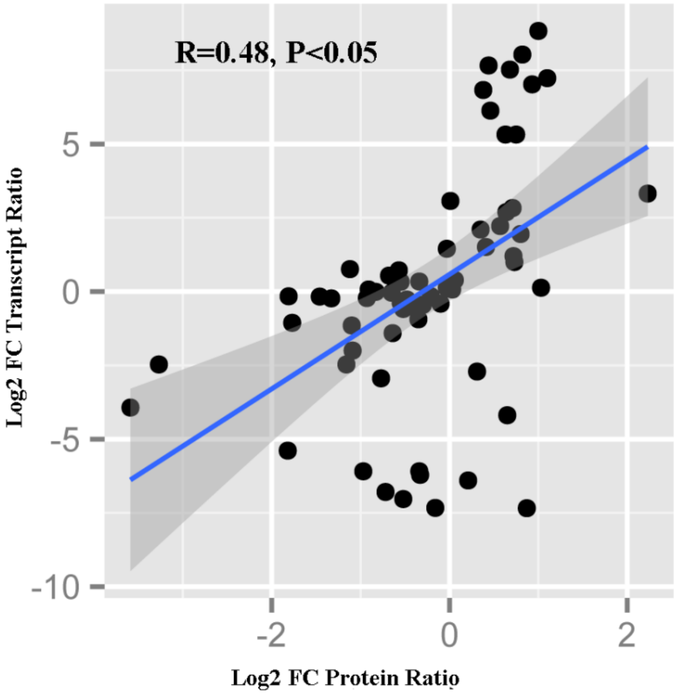

Figure S8. Concordance between changes in the protein and corresponding transcripts.

Table 1. Summary of sequencing data and alignment statistics (1, 2, 3 represented repeat 1, 2, 3, respectively).

Libray	No. of raw reads	Total length (bp)	Total bases (trimmed)	Total length (bp) of high-quality reads	Mapping ratio % (≤ 3 bp mismatch)	Mapping ratio % (≤ 2 bp mismatch)
**1-L0**	23314262	2331426200	19,915,018	1,737,915,587	91.07	89.18
**2-L0**	14588714	1415105258	12,092,374	1,060,319,827	80.55	75.77
**3-L0**	16291476	1580273172	13,306,428	1,144,643,922	81.39	76.68
**1-L1**	16291392	1629139200	14,184,838	1,220,133,252	93.89	92.7
**2-L1**	19893454	1929665038	16,461,692	1,415,549,817	82.3	77.71
**3-L1**	11424758	1108201526	9,568,666	826,388,750	81.64	77.19
**1-L2**	23885568	2316900096	20,098,070	1,674,574,399	92.71	91.54
**2-L2**	13408704	1300644288	11,154,754	966,968,998	81.26	76.53
**3-L2**	16328340	1583848980	13,508,604	1,164,116,872	81.03	76.27
**1-L3**	20567834	2056783400	18,028,992	1,554,070,513	92.05	90.64
**2-L3**	16266184	1577819848	13,485,326	1,166,389,856	81.06	76.34
**3-L3**	14418964	1398639508	11,853,156	1,022,707,425	81.33	76.62

Table S2. Statistics of annotation results for *D. latiflorus* unigenes.

Sequence file	All	NR	UniprotKB	GO	KEGG
**L0_L1-unigene**	3179	3045	2552	1861	1289
**L0_L2-unigene**	1732	1670	1415	988	709
**L0_L3-unigene**	4376	4129	3430	2478	1719

Table S3. Primers pairs used for Quantitative Real-Time PCR.

Gene ID	Description	Primer	
		Forward (5′- 3′)	Reverse (5′- 3′)
Unigene49490	MADS-box transcription factor 50-like	GTCGCGCTCGTCGTCTTC	ACATTGTCGGTATCGGCTGAT
Unigene57034	zinc finger CCCH domain-containing protein 55-like	AGGGCGAGGTAAAGGCAGAA	AGCAGGTCCTCCGGATGTT
Unigene108692	flower development related protein	GGTCGCGCTCATCGTCTT	CGATTTGCCTGGTCTCCAAT
Unigene32545	Ethylene-forming enzyme	TCGCTCTCCATGGACTTGAAG	GGCGTATCCCAAGTTCGTGTT
Unigene114564	zinc finger protein CONSTANS-LIKE 13-like isoform X2	GGGTCCCCGAGTTTGAGAAG	GGAAGCCATGGCAGGAAGT
Unigene120387	putative 29 kDa ribonucleoprotein A, chloroplast precursor	AGCCGTGGATTTGGATTCG	CCCTCAGAGCTCTTCCTTGAAA
Unigene103892	light harvesting chlorophyll a-b binding protein 2-1	CGGAGATCCTGTCCAAGA	GGTAAACCTTGTCGAGCC
Unigene104205	heat shock protein 70	CTTCTCCACCTACTCGGACAAC	CTCAAACTTGCCCAGCAGGTT
Unigene109097	ribulose bisphosphate carboxylase/oxygenase activase, chloroplastic-like	CAGCGAGTTCCCTGACTCAATC	CCAAACCAAGCTCTGGACTTCT
Unigene110315	protein TRIGALACTOSYLDIACYLGLYCEROL 2, chloroplastic-like	AGGATGAAAGGACAGCAAGGAG TA	TGCAACTTTTTCGGCCAACT
Unigene130058	chlorophyllase-2, chloroplastic-like	CGGAGATGGACACCTTGGT	GCCCTCAGCTTTACACGATAT
Unigene137345	17.8 kDa class II heat shock protein-like	TGTCCATGTCGGCGTTCTC	TCAAGGTGCAGGTGGAGGA
Unigene30387	photosystem I reaction center subunit IV, chloroplastic-like	GATCCGTCGTCACAGTTGAT	GTCCAGGGCGTAGTTGTTG
Unigene32039	chlorophyll a-b binding protein CP24 10B, chloroplastic-like	GCTTGCCAATGCCTCATCAGT	TGAACTCGGCGTCGCTCTT
Unigene33763	putative UVB-resistance protein (UVR8)	TGATTGCTGCTGGTGCTGAG	CGCCATCCACATGCAACAAG
Unigene36923	probable protein arginine N-methyltransferase 6.1-like	GGCACTGGGCTACGATCATC	TCACGACCCTCAATCTCAACCT
Unigene39652	MADS15 protein	CTGAAGCGGATCGAGAACAAG	CGTCGCAGAGGACGGAGAT
Unigene28224	cytokinin receptor CRE1b, putative, expressed	AGTTCCTTGCAACTGTTTCTCATG	GGGTTGTCTTTAGTTCTGTGCCTA A
EF1α	Reference gene	GTTGTTACCTTTGGTCCCAGCGG	TGGCAGGGTCATCCTTGGAGTTG

Table S4. Expression of the same clump of *D. latiflorus* flowering biomarkers in floral transition. FPKM, fragments per kilobase of exon per million fragments mapped. Genes with ratio values less than unity show increased expression during early stages of floral development; genes with ratio values greater than unity show increased expression during later stages.

GENE ID	Function discripsion (GO)	e-value	FPKM (L1)	FPKM (L2)	FPKM (L3)	L2:L1	L3:L2
**Continue increase in the gene expression**							
Unigene39652	MADS14 protein	3.00E-86	0.6	9.84	43.1	16.4	4.38
Unigene138210	loosening and extension of plant cell walls	e-150	0.06	3.19	13.7	53.17	4.29
Unigene43312	loosening and extension of plant cell walls	e-145	0.2	3.46	15.56	17.3	4.50
Unigene10723	Regulates floral architecture and leaf development	4.00E-55	0.01	0.67	3.32	67	4.96
Unigene114865	Probable transcription factor involved in plant development PREDICTED: scarecrow-like protein 14-like [Setaria italica]	0	0.01	0.48	2.5	48	5.21
Unigene111037	Abscisic acid 8'-hydroxylase 3	6.00E-11	0.01	0.34	6.8	34	20
**Continue decrease in the gene expression**							
Unigene38555	Promotes plant stress tolerance such as heat, chilling, salinity and methylviologen	6.00E-67	39.36	3.31	1.2	0.08	0.36
Unigene41907	FAR1-RELATED SEQUENCE 5	5e-25	1.98	0.19	0.01	0.10	0.05
Unigene21870	Sugar transport protein 11	2e-10	16.7	2.67	0.01	0.16	0.004
Unigene49486	Regulates shoot apical meristem (SAM) initiation and maintenance	9e-24	1.46	0.54	0.01	0.37	0.02

Unigene112435	negatively regulates salicylic acid-(SA-) dependent defense responses, abscisic acid (ABA) signaling, and ethylene-induced senescence	3e-21	1.31	0.36	0.05	0.27	0.14
Unigene133805	Modulates plant transpiration efficiency	0	1.23	0.28	0.01	0.23	0.04
Unigene14850	Transcription factor bHLH13	6e-67	2.97	0.82	0.36	0.28	0.44
Unigene14377	Glutamate synthase 1 [NADH], chloroplastic	0	0.82	0.29	0.05	0.35	0.17
Unigene137525	Outer envelope protein 61, chloroplastic	0	4.8	1.71	0.89	0.36	0.52
Unigene12963	C-terminal processing peptidase, chloroplastic	2e-65	1.14	0.48	0.15	0.42	0.31
Unigene99180	Involved in the regulation of stomatal aperture	0	0.98	0.38	0.01	0.39	0.03
Unigene27191	May be involved in oxidative stress response	e-113	10.81	6.13	2.41	0.57	0.39

Table S5. The pathway and the products involved in the pathway of plant hormone signal transduction.

**Pathway**	**Product**	**Pathway ID**
Tryptophan metabolism	Auxin/IAA	Ko00380
Zeatin biosynthesis	Cytokinin	Ko00908
Diterpenoid biosynthesis	Gibberellin	Ko00904
Carotenoid biosynthesis	Abscisic acid	Ko00906
Cysteine and methionnine metabolism	Ethylene	Ko00270
Brassinosteroid biosynthesis	Brassinosteroid	Ko00905
α-Linolenic acid metabolism	Jasmonic acid	Ko00591
Phenylalanine metabolism	Salicylic acid	Ko00360
